# MEG evidence for conceptual combination but not numeral quantification in the left anterior temporal lobe during language production

**DOI:** 10.3389/fpsyg.2014.00524

**Published:** 2014-06-04

**Authors:** Paul Del Prato, Liina Pylkkänen

**Affiliations:** ^1^Department of Psychology, New York UniversityNew York, NY, USA; ^2^Department of Linguistics, New York UniversityNew York, NY, USA; ^3^NYU Abu Dhabi Institute, New York University Abu DhabiAbu Dhabi, UAE

**Keywords:** cognitive neuroscience of language, syntax, semantics, language production, anterior temporal lobe, conceptual representation, quantification, magnetoencephalography (MEG)

## Abstract

The left anterior temporal lobe (LATL) has risen as a leading candidate for a brain locus of composition in language; yet the computational details of its function are unknown. Although most literature discusses it as a combinatory region in very general terms, it has also been proposed to reflect the more specific function of conceptual combination, which in the classic use of this term mainly pertains to the combination of open class words with obvious conceptual contributions. We aimed to distinguish between these two possibilities by contrasting plural nouns in contexts where they were either preceded by a color modifier (“red cups”), eliciting conceptual combination, or by a number word (“two cups”), eliciting numeral quantification but no conceptual combination. This contrast was chosen because within a production task, it allows the manipulation of composition type while keeping the physical stimulus constant: a display of two red cups can be named as “two cups” or “red cups” depending on the task instruction. These utterances were compared to productions of two-word number and color lists, intended as non-combinatory control conditions. Magnetoencephalography activity was recorded during the planning for production, prior to motion artifacts. As expected on the basis of comprehension studies, color modification elicited increased LATL activity as compared to color lists, demonstrating that this basic combinatory effect is strongly crossmodal. However, numeral quantification did not elicit a parallel effect, suggesting that the function of the LATL is (i) semantic and not syntactic (given that both color modification and numeral quantification involve syntactic composition) and (ii) corresponds more closely to the classical psychological notion of conceptual combination as opposed to a more general semantic combinatory function.

## INTRODUCTION

A fundamental question for the science of language is how general processes such as lexical access and composition decompose into specific computational subroutines. As regards the combinatory operations of language, brain research on sentence processing has in recent years progressed from broad characterizations of networks of regions implicated for the composition of sentences (e.g., Mazoyer et al., 1993; Just et al., 1996; Xu et al., 2005; Brennan and Pylkkänen, 2012) to the finding that among these regions, the left anterior temporal lobe (LATL) is consistently involved even in the most basic combinatory operations, such as building a small two-word phrase (Bemis and Pylkkänen, 2011a, 2013a,b). This allows us to ask a computationally more specific question: what aspect of basic composition is the LATL responsible for?

In the present work, we used magnetoencephalography (MEG) and a simple language production task to narrow down the hypothesis space regarding LATL function. Specifically, we asked whether the LATL is computationally specialized for the combination of conceptually rich content words, which have constituted the stimulus material of prior basic composition studies (Bemis and Pylkkänen, 2011a,2013a,b; cf., also Baron and Osherson, 2011), or whether its function extends to cases of numeral quantification, which would suggest a more general combinatory role. Under the first hypothesis, the function of the LATL would correspond closely to the classical notion of “conceptual combination” in Psychology (e.g., Hampton, 1997), whereas a more general computational profile would fit either syntactic or semantic composition as conceived of in theoretical Linguistics, where both types of composition apply across the board to all meaningful elements. The main advantage of using production was that it allowed us to vary composition type while keeping the physical stimulus constant: subjects named pluralities of colored objects (e.g., a picture of two red cups) either using color modifiers (e.g., *red cups*) or numeral quantifiers (*two cups*), depending on task instruction. The millisecond time resolution of MEG allowed us to a capture the planning stages of speech output before the onset of articulation-related motion artifacts. Crucially, behavioral evidence has shown that the conceptual and grammatical encoding of short two-word utterances such as those produced in our study is completed before articulation begins (Meyer, 1996; Schriefers et al., 1999; Alario et al., 2002). This fact combined with the high temporal resolution of MEG allowed us to obtain a detailed spatio-temporal map of the relevant conceptual and combinatory processes elicited by the planning of our two types of noun phrases.

The LATL was first implicated as a potentially central combinatory region in language in a series of hemodynamic studies employing structured sentences as the combinatory stimulus and unstructured lists of words as the non-combinatory control (Mazoyer et al., 1993; Stowe et al., 1998; Friederici et al., 2000; Vandenberghe et al., 2002; Humphries et al., 2005, 2006; Jobard et al., 2007; Rogalsky and Hickok, 2009; Pallier et al., 2011). A consistent finding from this set of auditory and visual comprehension studies was the increased involvement of the LATL in processing sentences as compared to lists. While an important initial discovery, the hypothesis space left open by this finding is still vast: on the basis of this result, the LATL could compute any aspect of the multitude of computations that contribute to sentence comprehension: syntactic composition, semantic composition, reference resolution, establishment of various types of dependencies, pragmatic inferencing, and so forth. To address the syntax vs. semantics question, several researchers have employed a so-called jabberwocky version of the sentence vs. list paradigm, substituting the open class items of the stimuli with pronounceable non-words, with the aim of engaging syntactic but not semantic processing with the non-word sentences (Mazoyer et al., 1993; Friederici et al., 2000; Humphries et al., 2006; Pallier et al., 2011). However, what a comprehender does with a stimulus such as *the solims on a sonting grilloted a yome and a sovir* (from Humphries et al., 2006) is a rather complicated question and subjects’ strategies could vary substantially. Given that many types of semantic information are still present in jabberwocky sentences – e.g., the just mentioned sentence clearly conveys that individuals belonging to the solim-category participated in a grilloting-activity that affected individuals belonging to the yome and sovir-categories – a motivated subject might work quite hard to extract this information. In contrast, a less motivated subject could easily process these rather boring stimuli in a very shallow, non-semantic way. Thus the degree to which jabberwocky sentences engage semantics is not obvious and indeed results from these manipulations have been somewhat mixed, with non-word sentences eliciting an LATL increase in several studies (Mazoyer et al., 1993; Friederici et al., 2000; Humphries et al., 2006), but not in all (Pallier et al., 2011). Thus, the syntax vs. semantics question regarding LATL function is still far from settled.

Several hypotheses left open by the sentence vs. word list studies have, however, been ruled out by a series of recent MEG studies employing minimal two-word phrases as the combinatory stimuli (Bemis and Pylkkänen, 2011a, 2012, 2013a,b). In these studies, adjective–noun combinations such as *red boat* were compared to non-combinatory one-word stimuli (*boat*) as well as to lists consisting of two nouns (*cup*, *boat*). The most consistent finding from this work was the increased amplitude in the LATL for the adjective–noun combinations as compared to the non-combinatory controls, an effect that was robust both to modality (Bemis and Pylkkänen, 2012, 2013b) as well as to task demands (Bemis and Pylkkänen, 2013a). This LATL effect was relatively early, peaking at 200–250 ms post-noun onset, preceding other, less stable effects in the ventromedial prefrontal cortex (vmPFC; Bemis and Pylkkänen, 2011a) and the angular gyrus (Bemis and Pylkkänen, 2012). Since these small two-word phrases do not involve many of the computations engaged by full sentences, arguably eliciting only the most basic forms of composition, these findings strongly suggest that the LATL effects observed in the sentence vs. word list studies in fact reflect very basic combinatorics as opposed to other, more sentence-level phenomena. Whether the LATL computes syntactic or semantic structure is of course still not determined as combining adjectives and nouns clearly involves the composition of both.

In addition to varying composition in a more targeted way, this minimal two-word paradigm translates well into a production task, as the syntactic and semantic planning of small two-word phrases is known to occur essentially entirely before the onset of articulation (Meyer, 1996; Schriefers et al., 1999; Alario et al., 2002). Given a technique with fine temporal resolution such as MEG, this provides the opportunity to characterize in great detail the computational steps of the various planning stages prior to the onset of the motion artifacts caused by mouth movement. In a production follow-up to the comprehension studies described above, Bemis and Pylkkänen (2011b, under revision) showed that the LATL basic composition effect observed in comprehension indeed generalizes to production: adjective–noun productions elicited a robust LATL increase compared to non-combinatory list controls. These MEG findings conform to prior results from PET showing that both narrative comprehension and production engage the LATL (Awad et al., 2007).

Although studying syntactic and semantic production is in many ways harder than studying their comprehension – the primary challenge being getting subjects to say what you want them to – a picture naming paradigm does provide one unique benefit over reading or listening, namely that an identical picture can be used to elicit different productions depending on the task instruction, providing a perfect control of perceptual processing (e.g., Indefrey et al., 2001, 2004). Here, we took advantage of this and asked subjects to produce either adjectival modifications (“red cups”) or numeral quantifications (“two cups”) in response to pictures depicting several colored objects. This simple set-up allowed us to address whether the basic composition effect observed in the LATL specifically reflects the combination of individual features into a more complex concept or also extends to the enumeration of individuals belonging to a certain category. As in many of our prior studies, and borrowing from the sentence vs. list literature, the non-combinatory controls were list productions, a list of two colors functioning as the control for the color modifications (“red, blue”) and a list of two numbers as the control for the numeral quantifications (“two, three”). Our goal in designing the list controls was for them to be maximally natural utterances despite being non-combinatory, i.e., lists of numbers and colors are in fact possible natural utterances.

An LATL combinatory effect for color modifications would not only conform to the literature cited above but also resonate with the so-called hub-and-spoke model of LATL function within the semantic memory literature (Patterson et al., 2007; Lambon Ralph et al., 2010). In this prominent model, supported by both neuroimaging (Gauthier et al., 1997; Grabowski et al., 2001; Bright et al., 2004; Tyler et al., 2004; Rogers et al., 2006; Clarke et al., 2011, 2013) and neuropsychological data (Hodges et al., 1995; Rogers et al., 2006), the LATL is considered a hub in which a distributed conceptual representation is bound together and processed by a common set of neurons. Although the semantic memory literature has been focused on single words and concepts, this hypothesis quite naturally extends to concepts conveyed over multiple lexical items (Westerlund and Pylkkänen, 2014): for a single concept such as “banana,” the LATL by hypothesis binds together the prototypical shape and color of bananas, whereas for a small phrase such as “red cup,” the LATL would similarly combine the “red” and “cup” features into a combined conceptual representation. In fact, a recent MEG study demonstrated that the LATL effect of adjectival modification on nouns is larger when the noun itself has a less specific meaning, suggesting that both variables affect the same neural response (Westerlund and Pylkkänen, 2014). A type of combinatory effect for single words was also demonstrated by Baron and Osherson (2011) who showed that the LATL activation elicited by a concept such as “boy” is correlated with the product of activations for concepts representing features that contribute to the meaning of “boy,” such as “male” and “child.” On the basis of this, Baron and Osherson (2011) concluded the LATL to be a central site for conceptual combination.

Although conceptual combination is often defined in very general terms as “the construction of complex concepts from simple constituents” (Baron and Osherson, 2011, p. 1847), research on conceptual combination has traditionally focused on composition among content words describing properties of individuals and, within this, the composition of nouns with adjectival and nominal modifiers, in particular. As a specific example of what “conceptual combination” is thought to cover, Hampton (1997) describes different “types of conceptual combination” as consisting of (i) intersective combinations of concepts such as “red apple,” (ii) non-intersective combinations of modifiers and nouns such as “criminal lawyer” (which in the dominant reading does not refer to individuals who are both criminals and lawyers), and (iii) lexicalized compounds such as “bull ring” with various degrees of idiomatic meaning. Thus, traditionally, research on conceptual combination has not investigated combinatory operations involving function words, such as the composition of phrases like “the dog,” “not happy” or “some boy.” This radically contrasts with the research foci in theoretical Linguistics, where the process of “semantic composition” is taken to cover every instance of two meanings combining in natural language (e.g., Heim and Kratzer, 1998) and, in fact, the majority of research effort is typically spent in examining the meanings of various functional elements, such as quantifiers, determiners, and other closed class items. Whether or not researchers within the Psychology tradition have intended “conceptual combination” to cover cases such as “three cups,” where “‘three” does not describe a property of cups but rather expresses that the set of cups has the cardinality 3, it is empirically quite possible that there is a special process of “conceptual combination” that is separate from the composition of more grammatical elements such as determiners, numerals, quantifiers, and so forth. If natural language did draw this distinction, it would be a fundamental computational division with important consequences for every theory of syntax and semantics. Our aim was to contribute an initial dataset from brain measurements speaking to this question.

As regards to the anatomical focus of our study, semantic effects within the LATL have in prior literature shown a fair amount spatial variability, which somewhat complicates the definition of a suitable region of interest (ROI) for our analysis. For example, Westerlund and Pylkkänen’s (2014) adjectival modification effect localized more laterally than the combinatory effects of both Bemis and Pylkkänen (2011a, 2013a) and Baron and Osherson (2011), the latter studies finding effects contained more or less within the temporal pole (BA 38). Within research on single concepts as conveyed by pictures or words, semantic effects have also occurred at least within the temporal pole (e.g., Gauthier et al., 1997), on the ventral surface of the anterior temporal lobe (Binney et al., 2010; Visser et al., 2012) and more medially (Clarke et al., 2011; Tyler et al., 2013). It should be noted though that the details of the stimulus manipulations and tasks vary across this literature making any direct comparisons complicated. For the purposes of the current study, we chose the temporal pole, BA 38, as the focus of our ROI analysis based on the original, two-word composition findings of Bemis and Pylkkänen (2011a, 2013a). Crucially, however, our ROI analysis was followed by uncorrected full brain contrasts allowing us to visualize in more detail the activation centers of any obtained effects.

The full brain analysis also allowed us to assess whether regions other than the LATL were robustly affected by our stimulus manipulation. While the LATL has in prior studies been the most consistent locus of basic composition effects within two-word phrases Bemis and Pylkkänen (2011a, 2013a,b), Westerlund and Pylkkänen, 2014), such effects have also been observed in the vmPFC (Bemis and Pylkkänen, 2011a, 2013a,b) and the angular gyrus (Bemis and Pylkkänen, 2012). While the participation of these regions in the brain’s general “meaning network” is well-established (e.g., Binder et al., 2009; Binder and Desai, 2011), it is specifically the LATL for which the particular computation of conceptual combination has been hypothesized (Baron et al., 2010; Baron and Osherson, 2011). Given that in our studies this region has also shown the greatest replicability with respect to composition effects within two-word phrases, we limited our ROI analysis to BA 38 to allow for a maximally powerful test of our hypotheses within this region. But importantly, our rather liberally thresholded full brain contrasts also enabled the visualization of any additional effects outside of our ROI.

## MATERIALS AND METHODS

### SUBJECTS

Eighteen right-handed, non-colorblind, native English speakers (12 female) with normal or corrected-to-normal vision participated in the experiment, 3 at our MEG facility in NYU Abu Dhabi, and 15 at NYU New York (average age: 23.6 years). All participants provided consent before participating in the experiment. Two participants were excluded from data analyses, one due to a noisy recording environment that resulted in the loss of over half of experimental trials, and one due to lack of sustained wakefulness during the experiment session. The data from the remaining 16 subjects were included in the final analysis.

### STIMULI AND DESIGN

**FIGURE 1:**
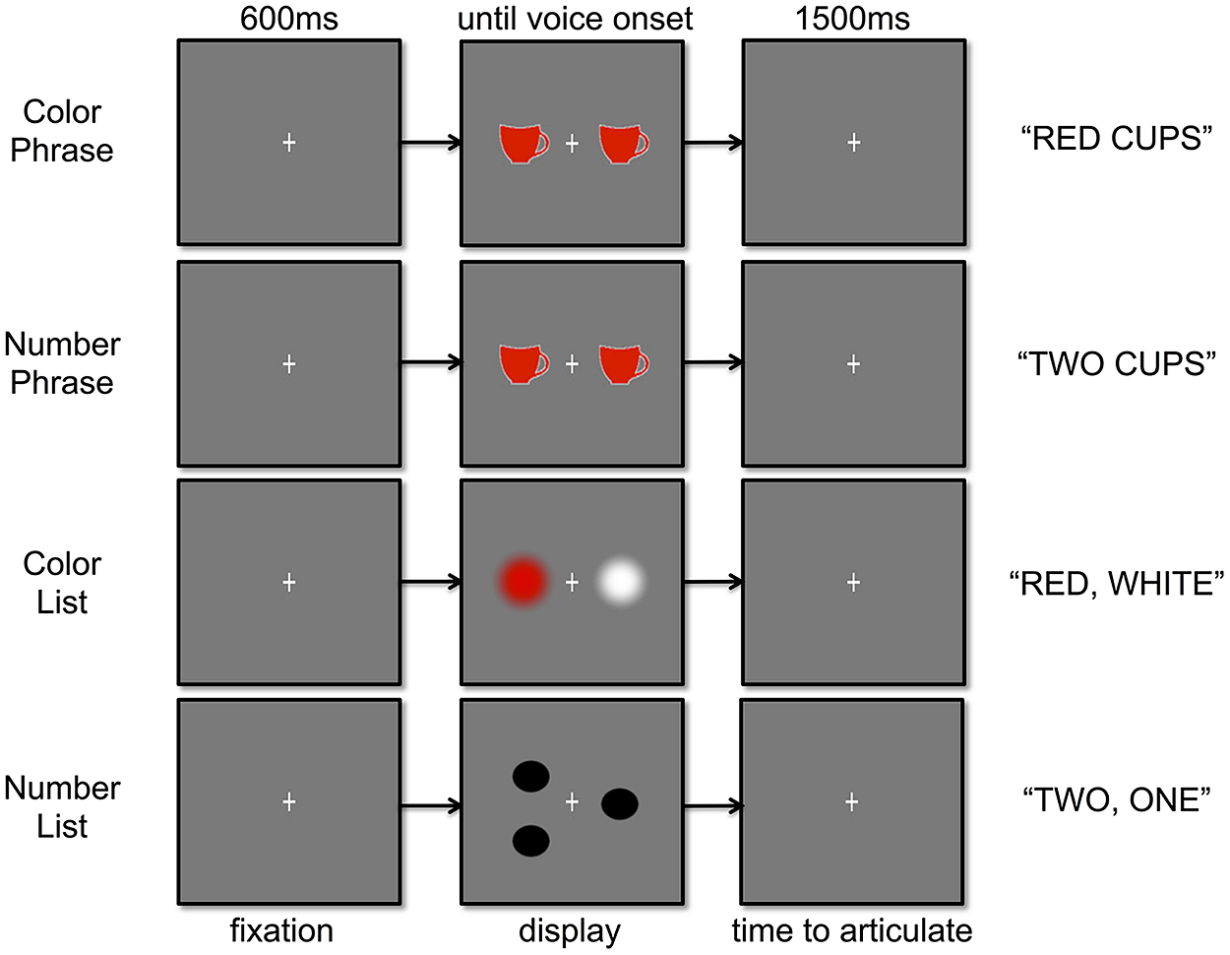
**Trial structure and experimental design**.

Our design employed a picture-naming task and aimed to isolate neural activity associated with utterance planning prior to the overt articulation (and resulting motion artifacts) of simple two-word strings. In the two main composition tasks, the participant was presented with a display of multiple colored objects and was instructed to describe either the color or the numerosity of the objects in a two-word noun phrase, such as “red cups” or “two cups” (**Figure 1**). An important requirement for this design was that the enumeration should not be harder than the color modification. Consequently, only numbers 1, 2, and 4 could be included, as numbers higher than this cannot be estimated quickly and automatically (Mandler and Shebo, 1982; Trick and Pylyshyn, 1994; Feigenson et al., 2004). The color words were chosen such as to match the number words as closely as possible while also being maximally visually distinctive from each other (if the latter constraint was not met, then color naming could have turned out harder than number naming). Consequently, we required the color words to be monosyllabic like the number words but for the rest of lexical-level variables, we chose English Lexicon Project naming times as a summary statistic (Balota et al., 2007). The colors *red*, *blue*, and *green* were chosen as optimally satisfying these objectives. Numerically, the English Lexicon Project naming times (Balota et al., 2007) of these color words were somewhat faster (mean = 552 ms) than those of the Number words (mean = 620 ms), but this difference was not significant (*p* = 0.12). Finally, the head noun following the number and color words was always one the following three plural nouns: *cups*, *boats*, and *locks*. These objects were chosen due to high visual discriminability, with the following linguistics properties: (1) monosyllabic, (2) bimorphemic (one morpheme in root, and –s), and (3) relatively constant log frequency as described by the HAL corpus (between 8.633 and 8.795; Balota et al., 2007).

The displays eliciting the color modifications and numeral quantifications consisted of a fixation in the middle of the screen, with at least one object placed on both sides of the fixation. To avoid eye movements, subjects were instructed to focus on the fixation while naming. To make this possible, the objects in the Phrase conditions as well as the control conditions described below were all placed close to the fixation and the entire display was rather small – objects in each visual display were no more than 2.5 cm from the fixation cross on either side. The presentation screen was 42 cm from the eyes of the participant, and thus objects on each side of the display occupied at most 3.5° of a participant’s field of vision.

Two control conditions aimed to generate two-word productions of color and number words in a non-combinatory manner. In the color list condition, the participant was instructed to name two color patches on opposite sides of the fixation cross, from left to right (i.e., “red, white”). The first color to be named was limited to the color modifiers also present in the composition task, to keep the first word of the utterance between composition and list conditions matched. The second color to be named was “pink,” “brown,” or “white,” i.e., distinct from those used in the color modification condition, to match the amount of lexical variability in the composition and list conditions. The naming times of the second position colors were matched to the naming times of the plural nouns in the composition condition (Color mean = 579 ms, Noun mean = 575 ms, *p* = 0.79).

Generating a parallel list condition for the number words faced the challenge of matching the amount of lexical variability in the Number Phrase and Number List conditions. This required us to use numbers higher than four in the second position – otherwise two, three, and four would have been employed in both positions, resulting in more lexical repetition in the Number List condition than the Number Phrase condition. As a compromise, we used “one” in second position, still allowing automatic enumeration, as well as “five” and “six,” but to alleviate the cognitive difficulty of these higher numerosities (Feigenson et al., 2004), we displayed all Number List stimuli as dice-like arrangements to generate a symbolic representation that did not require counting (see **Figure 1**). Our reaction time data, showing no delay for the Number List condition, suggest that this strategy indeed worked for making these larger numbers easier. The left arrangements were designed to elicit identical responses as the numeral words in the Number Phrase condition (two, three, four). Corpus naming times of the right arrangements (one, five, six) were matched to the plural nouns (Number mean = 588 ms, Noun mean = 575 ms; *p* = 0.65).

### PROCEDURE

The experiment contained 360 trials, with 90 trials in each condition. As each condition was devised to have ninepossible utterances (3 options for first word^*^3 options for second word), the stimuli were repeated 10 times to reach this total. The stimuli were blocked by condition, with 18 trials presented in each block. Prior to a block, a break period was given and participants pressed a button to continue the experiment. Instructions (for each corresponding condition – “Name the color and objects”; “Name the total number and objects”; “Provide both color names”; “Provide both numeral names”) were provided before each block presentation to notify the participant of the upcoming condition. The order of the condition blocks was randomized, with the constraint that each cycle of conditions occurred before the next (i.e., the first blocks of each condition occurred in a random order, then the second blocks, and so on). Between trials, a constraint was placed in the randomization such that no stimulus eliciting a particular utterance (e.g., “red cups”) would elicit that same utterance in the next trial.

Participants were instructed to maintain their gaze on a fixation cross, consistent for the duration of each experimental block, to limit eye movements. To avoid blink artifacts during the critical analysis window, subjects were asked to not blink while the picture was on the screen. Instead, participants were to blink between trials, i.e., after articulation but before the next picture. Also, blinking was encouraged during the break periods.

For each trial a visual display of the stimulus appeared on the screen until voice onset. Voice onset was triggered via a microphone set-up approximately 6 ″ from the participant’s mouth inside the MEG. Prior to data acquisition, the voice onset trigger activation level was always adjusted to a high enough threshold such that only clear overt naming responses activated it, as opposed to sounds such as lip-smacking. A later comparison with saved sound data was used to verify that this was indeed successful. Upon voice onset, the image disappeared, which allowed us to time the experiment at a comfortable rate for each individual (as opposed to keeping the stimulus on for a fixed amount of time in across the board). After voice onset, audio data was recorded for the next 1.5 s to acquire response information. An additional inter-trial interval of 0.5 s ±SD of 1s occurred prior to the presentation of the next trial. The MEG experiment lasted approximately 25 min.

Prior to this main task, participants were familiarized with each image type used in the experiment and were provided with the intended name. A practice session was conducted outside the MEG room with no microphone set-up. The individual stated their response for each trial, to the experimenter’s acknowledgment, and the participant pressed a key to simulate voice onset to continue to the next trial. The practice experiment was a heavily abbreviated version of the actual task.

Headshape information for each participant was collected using a Polhemus Fasttrak 3D digitizer, and five marker coils were placed around the head to be co-registered with the location of the MEG sensors prior to source localization. During the experiment, participants laid in a dimly lit, magnetically shielded room. Stimuli were presented using Psychtoolbox (Brainard, 1997; Pelli, 1997) on a neutral gray background. Magnetic field activity was recorded using an axial gradiometer array (Kanazawa Institute of Technology), with 157 sensors (New York) or 208 sensors (Abu Dhabi).

In total, the entire experimental session lasted approximately 90 min.

### DATA ANALYSIS

Magnetoencephalography data were collected continuously, noise-reduced using the Continuously Adjusted Least Squares Method (CALM; Adachi et al., 2001), and then epoched for each trial starting 200 ms prior to the onset of the visual display, and continuing 700 ms post-stimulus onset. Artifacts were removed from the data by rejecting trials where the maximum amplitude during the epoch length exceeded 3000 fT, when an eyeblink artifact was present during the epoch (determined by visual inspection of maximum eye blink sensors), or when large noise bursts were detected in the data. Furthermore, trials with response times faster than 300 ms and slower than 1500 ms were excluded as outliers from both behavioral and MEG analyses. This resulted in the loss of 29.9% of trials.

Raw data were high-pass filtered at 1 Hz prior to subsequent analysis steps. Source activity was estimated using distributed L2 minimum norm estimates (Hämäläinen and Ilmoniemi, 1994) for each subject and each averaged condition in BESA (version 5.1.8.10; MEGIS Software, GmbH). A low-pass filter was then applied at 40 Hz. The baseline, used as the noise covariance matrix in the computation of minimum norm estimates, was defined as the 100 ms prior to the onset of the stimulus. To assess the consistency of the minimum-norm estimate values between conditions, a repeated-measures 2 × 2 ANOVA [Word Type (Color vs. Number) × Composition (Phrase vs. List)] and follow-up pairwise *t*-tests were conducted on the signal-to-noise ratios of the minimum-norm estimates (Color Phrase *M* = 3.91, Number Phrase *M* = 3.57, Color List *M* = 3.34, Number List *M* = 4.10). Results showed no reliable main effects or interaction nor any significant effects in pairwise comparisons.

Region-of-interest (ROI) analyses were conducted using Brodmann area (BA) 38, covering the left temporal pole. Brodmann area labels were assigned to the 713 sources on the BESA-averaged brain using the Talairach Daemon (Lancaster et al., 2000) on the basis of the source coordinates in Talairach space.

The effect of the experimental manipulation on BA 38 activity was assessed with a cluster-based permutation test (Maris and Oostenveld, 2007) aimed at identifying temporal clusters of activity that were significantly affected by our experimental manipulation, corrected for multiple comparisons. As the test statistic, a 2 × 2 ANOVA was used with the factors Word Type (Color vs. Number) and Composition (Phrase vs. List). For initial cluster selection, we required an uncorrected *p*-value of 0.3 as in Bemis and Pylkkänen (2011a, 2012, 2013a,b). Next, a cluster-level statistic was calculated by summing test statistics from contiguous milliseconds that fell below the threshold. If a point-wise statistic did not fall below the threshold, then the cluster ended at the previous time-point. The cluster with the largest summed test statistic was isolated, and from 10,000 random permutations, a corrected *p*-value was generated as the ratio of permutations yielding a higher test statistic than the actual observed test statistic. The permutation test was conducted at the mid-latency time interval of 100–400 ms, aimed at excluding very early perceptual effects as well as late activity reflecting any aspect of motor planning and/or execution, including associated artifacts.

To complement the ROI analysis, uncorrected pairwise *t*-tests over the full brain were performed to verify that observed ROI effects in fact reflected activity within BA 38, as opposed to spillover activity from neighboring regions. In this analysis, spatiotemporal clusters were required to remain significant (*p* < 0.05) for at least 10 ms and over at least three adjacent source locations.

Finally, we tested whether any observed BA 38 effect was accompanied by a parallel effect in the MEG sensor data, free of source modeling assumptions. The left frontal quadrant of the sensor array was included in this analysis, with the aim of capturing activity generated in BA 38. For increased spatial sensitivity, this quadrant was further divided into anterior and posterior sections. Root mean square calculations of the signal at each sensor were averaged within the partitions and submitted to a 2 × 2 cluster-based permutation ANOVAs identical in all parameters to the one described above for the ROI analysis. Results in the two partitions were also FDR corrected (Genovese et al., 2002) with a criterion value of 0.05. The aim of our sensor analysis was to provide a maximally simple and easily replicable assessment of our hypothesis, although the roughness of this analysis, i.e, the use of the same sensors across all participants despite different head locations in the helmet, obviously lessened our chances of closely mirroring the source analysis.

## RESULTS

### BEHAVIORAL RESULTS

Mean naming latencies by condition were analyzed with a 2 × 2 repeated-measures ANOVA (Word Type by Composition). Response times shorter than 300 ms were omitted from this analysis due to microphone voice onset malfunction (note: our ROI analysis includes data up to 400 ms, but response times between 300 and 400 ms comprised only 6.2% of all trials). Additionally, responses greater than 1500 ms were excluded as outliers (>2 SD from the mean across subjects). In sum, a total of 14.5% of reaction times were excluded from analysis. Results on the remaining data showed no main effects and no interaction of Word Type by Composition (all *F*’s < 1; mean ± standard deviation for each condition – Color Phrase: 782 ms ± 284ms, Color List: 777 ms ± 283 ms, Number Phrase: 740 ms ± 254 ms, Number List: 717 ms ± 240 ms). This suggests that the conditions were of similar cognitive difficulty, and that each required a relatively constant amount of effort to plan and utter the desired response.

During data acquisition, it was found that participants’ responses were correct at ceiling levels (near 100%), presumably due to sufficient practice of the task prior to the MEG recording and the presence of item-based repetition in the experiment. Consequently, we forewent analysis of accuracy data and did not reject trials solely on the basis of accuracy. Additionally, in the uncommon case of an error, the linguistic structure of the given utterance always mirrored the structure of the desired response and therefore retained the type of processing intended for the particular condition.

### ROI RESULTS

**FIGURE 2:**
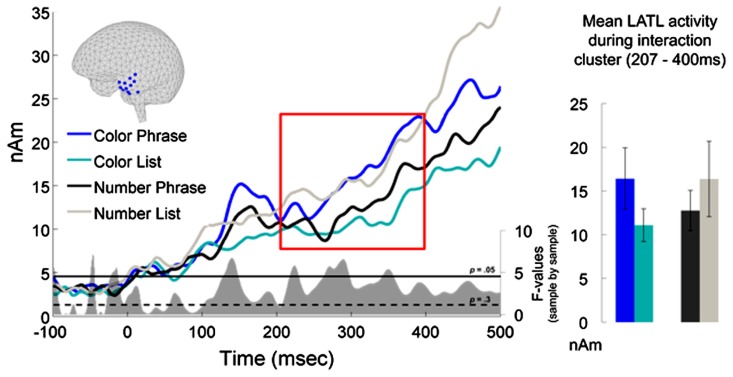
**ROI results for left temporal pole, BA 38.** The sources plotted in blue on the cortical surface were assigned to BA38 using the Talairach Deamon. The red box indicates the interval during which the interaction between Word Type and Composition was significant, Color Phrases eliciting higher activation than Color Lists, while the corresponding Number conditions patterned in the opposite direction. At the bottom of the waveform graph, the uncorrected *F*-values of the interaction are plotted in gray for each millisecond, along with horizontal lines indicating *p*-values of 0.05 and 0.3, the latter of which served as the criterion for cluster inclusion. On the right, activation means and standard errors are plotted for the interval where the interaction was observed.

**Figure 2** plots the time course of left BA 38 activation for the four experimental conditions. No reliable main effects were observed, but the cluster-based permutation test identified a significant Word Type × Composition interaction in a cluster extending from 207 to 400 ms (*p* = 0.046). Planned pair-wise comparisons showed this interaction to be driven by a reliable increase in activation for the Color Phrases as compared to the Color Lists (212 ms–400 ms; *p* = 0.035, two-tailed) while no parallel effect was observed for the Number Phrases as compared to the Number Lists (one very early cluster was identified at 100–129 ms, with larger amplitudes for Number Lists than Number Phrases, but it did not approach significance, *p* = 0.6). A direct comparison of Color Phrases and Number Phrases also revealed a cluster of increased activity for Color Phrases at 253–400 ms, although this difference was only marginal after correction for multiple comparisons (*p* = 0.1, two-tailed). Importantly, the increase observed for Color Phrases over Number Phrases cannot be interpreted as reflecting preferential processing of simply color words over number words since the Number Lists trended toward higher amplitudes than Color Lists. Also, although the increase for Color Phrases lasted beyond 300 ms, our cut-off for excluding trials with fast behavioral responses, it should be noted that only 6.2% of reaction times fell into the 301–400 ms range.

**FIGURE 3:**
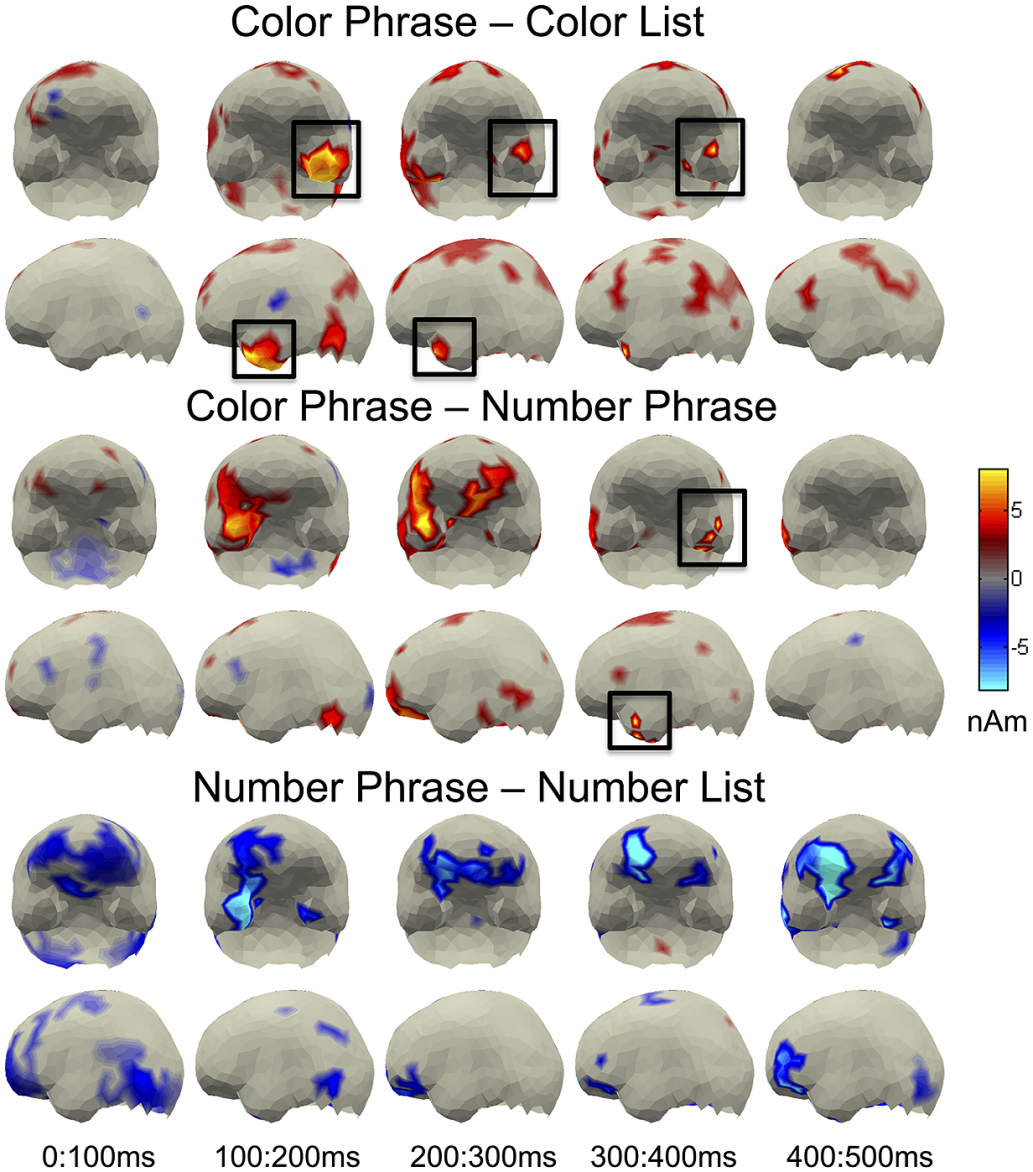
**Whole brain contrasts for pairwise comparisons**. LATL effects corresponding to the ROI results are indicated with black boxes.

To better understand the temporal distribution of our effect, the interaction *F*-statistic used for cluster selection was visualized sample-by-sample after the permutation test (**Figure 3**). This revealed an additional early peak at around ~150ms which was analyzed *post hoc*. The permutation test was ran in a narrow, early time window of 100–200 ms, but the effect was only marginal despite the very targeted analysis (*p* = 0.078).

In sum, the ROI results replicate in production the prior comprehension result that the LATL is sensitive to adjectival modification (Bemis and Pylkkänen, 2011a, 2012, 2013a,b). Additionally, we show that this effect does not appear to extend to numeral quantification, suggesting that the computational role of the LATL is more specialized than general semantic or syntactic composition.

### FULL BRAIN RESULTS

Uncorrected pairwise full brain comparisons were conducted between both Phrase conditions and their List controls as well as between the Color and Number Phrases (**Figure 3**). In **Figure 3**, significant activity at the *p* < 0.05 level is shown, with a color-scale plotting amplitude differences, in nAm, of the source estimates that showed a significant difference. The Color Phrase > Color List comparison conformed to the ROI results in showing a sustained LATL increase lasting until approximately 450 ms post-stimulus onset. No corresponding effect was observed for the Number Phrase > Number List comparison; in fact, activity appeared greater in the Number List condition in fronto-temporal regions of the right hemisphere. This may reflect that despite the absence of response time delay for Number Lists, this condition may still have induced some degree of added cognitive load. However, crucially, a LATL increase can be observed for the Color Phrases as compared to the Number Phrases, elicited by the same visual stimulus, conforming to the ROI findings.

Additionally, a large increase in the same direction was observed in the right anterior temporal lobe, suggesting that ATL sensitivity to color modification over numeral quantification may in fact be bilateral. Since the LATL effects of many sentences vs. word list studies have also been accompanied by a parallel though usually weaker effect in the right hemisphere (e.g., Stowe et al., 1998; Friederici et al., 2000), we explored whether this effect would survive correction for multiple comparisons in a *post hoc* ROI analysis on sources assigned to the right BA 38. However, the Color Phrase vs. Number Phrase contrast yielded no clusters approaching significance. Thus although smaller, the left BA 38 increase for Color Phrases over Number Phrases was statistically more reliable than the corresponding effect in the right hemisphere. In addition, the whole brain contrast on Color Phrases vs. Number Phrases showed increased activity for Color Phrases in vmPFC. Although the vmPFC is sensitive to semantic composition (as reviewed in Pylkkänen et al., 2010), including basic composition (Bemis and Pylkkänen, 2011a), this difference was taken to fall outside the hypothesis space of the current study and was not explored further. All other effects observed in the full brain analysis were weak and spatially fractionated, including activity around the angular gyrus – commonly thought of as part of “semantic network” (e.g., Binder et al., 2009; Binder and Desai, 2011) – showing a trend toward larger amplitudes for Color Phrases over Color Lists, but no corresponding effects for the same contrast in the Number stimuli. As regards possible increases for Number Phrases over Color Phrases, no robust effects were observed. The region most typically associated with processing of numerosities, the intraparietal sulcus (Dehaene et al., 2003), showed some increase for Number Lists over Number Phrases in an early time window (100–200 ms), potentially due to the presence of two numbers in the lists (as opposed to only one in the phrases).

### SENSOR RESULTS

**FIGURE 4:**
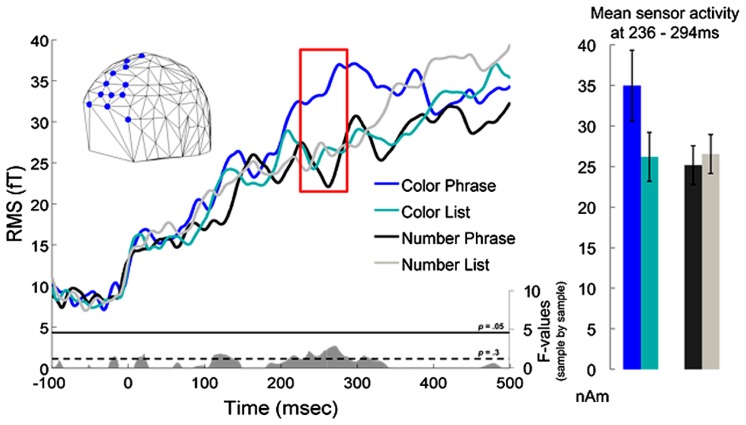
**Sensor results for the left anterior octant of the sensor array.** As in the ROI results, Color Phrases elicited higher activation than Color Lists, while no similar difference was observed for the Number conditions. The red box indicates the interval during which a marginal interaction cluster was observed (236–294 ms). An embedded graph depicts interaction *F*-values at each millisecond in gray, with thresholds for *p*-values of 0.3 and 0.05 included. To the right, condition averages and standard errors are plotted for the highlighted time window.

The sensor analysis indicated a waveform separation in the left anterior quadrant that was qualitatively similar to the interaction seen in the left BA 38 ROI analysis. This waveform separation corresponded to an interaction cluster in the 2 × 2 ANOVA in the more anterior partition of the quadrant at 236–293 ms, but the cluster did not survive correction for multiple comparisons (*p* = 0.087) (nor did it, in fact, ever show point-by-point uncorrected significance at the “standard” 0.05 level). Although not significant, the sensor results are nevertheless plotted in **Figure 4** for descriptive purposes: given this pattern, it is possible that with more subjects one could study the effect we report using only sensor data. It is important to keep in mind though that the sensor activity is likely to reflect activity from many anterior regions, and is thus unlikely to crisply correlate with any one particular effect in source space.

## DISCUSSION

In this work, we employed a simple language production paradigm to investigate the computational limits of the LATLL, implicated for syntactic and semantic processing by a large body of literature. Participants named pictures of colored objects using two-word phrases that described either the color of the objects (“red cups”) or the number of tokens displayed (“two cups”). Although both types of phrases involve the construction of syntactically and semantically complex phrases from smaller parts, only the former, “red cups,” is an example of “conceptual combination” as this term is typically used in the psychological literature on concepts. Our results show a combinatory effect in the LATL only for the color modifications compared to their non-combinatory controls and none for the numeral quantifications. Thus our results suggest that the role of the LATL may indeed be limited to conceptual combination without extending to other types of semantic composition, such as the composition of elements with more functional, as opposed to conceptual, meanings. In terms of timing, the onset of our conceptual combination effect at around 200ms was cotemporaneous with timing estimates of lexical and conceptual access in single-word electrophysiological studies (Indefrey and Levelt, 2004). Given that psycholinguistic models suggest syntactic information to become accessible with the lemma (Levelt, 1999; Ferreira, 2000), our effect timing conforms well to predictions arising from this combination of prior behavioral and electrophysiological work.

At the lexical level, the general distinction between content vs. function words is typically taken to be a fact about the grammar – albeit a fuzzy one (Friederici, 1982; Bebout, 1993) – supported by results from adult psycholinguistics (?), language acquisition (Bates et al., 1994; Caselli et al., 1995), and aphasia (Goodglass et al., 1972). However, whether combinatory operations between function and content words differ is a separate, so far mostly uninvestigated question, with the present results providing some initial evidence that a contrast may exist. Sentence vs. list studies using jabberwocky stimuli also speak to this issue, as jabberwocky sentences could be construed as engaging function word composition only. Since several studies using jabberwocky sentences have observed an LATL increase for such stimuli (Mazoyer et al., 1993; Friederici et al., 2000; Humphries et al., 2006), there is an interesting tension between the present results and this hemodynamic literature. Critically, jabberwocky sentences always include a wide variety of function words, leaving it unclear which word types might be driving an observed LATL effect. In contrast, the present study only compared the composition of two-word classes, color-adjectives, and number words, and thus future research will need to assess how our findings generalize to other word types. Number words are also in many ways special, sharing some, but not all, distributional properties with adjectives and quantifiers (Bloom and Wynn, 1997) and exhibiting some, but not all, prototypical characteristics of function/grammatical words (e.g., Keizer, 2007). Thus the critical question for future research is characterizing the true generalization that matters for LATL activation across a wide variety of word types varying in syntactic and semantic properties. In the best-case scenario, such research should provide a neurobiological definition of “conceptual combination.”

Since here we found no evidence for numeral quantification in the LATL, an important question left open by the current results is where and when the composition of number phrases occurs. We explored this in our whole brain analyses, but no region appeared to elicit increased activity for Number Phrases over Number Lists, suggesting that despite our efforts, the Number Lists may have ended up too difficult to truly function as a successful control condition. Given the less than perfect spatial resolution of MEG, a small positive effect could easily go undetected in the presence of strong opposing effects in neighboring regions, potentially reflecting general difficulty. Thus future studies on number phrase composition should aim to find a more straightforward non-combinatory control stimulus. Here we observed that the LATL preferentially computes color modifications over numeral quantifications, but given the concerns about the Number List control condition, the stronger conclusion that the LATL is not at all engaged by numeral quantifications cannot yet be drawn.

The whole brain contrasts also revealed that the increase for Color Phrases over Number Phrases was not only localized in the LATL, but also occurred in the right anterior temporal lobe, vmPFC and, more weakly, in more posterior temporal regions. Thus, it is possible that a rather extended network of regions preferentially computes conceptual combination-type processing as opposed to numeral composition. Given that the Color and Number Phrases were both elicited with the same physical stimulus, these differences cannot be attributed to processes relating to the semantic “comprehension” of images – presumably in both cases the participants conceptually processed the image as a plurality of colored objects but then, depending on condition, constructed different messages for naming it.

## CONCLUSION

To summarize, this study investigated the computational generality of the LATL by testing (i) whether LATL effects of adjective composition, previously shown for comprehension, would also be elicited in language production and (ii) whether such an effect would extend to the composition of number phrases. The adjective composition effect was straightforwardly replicated, suggesting a shared combinatory machinery between comprehension and production, but no LATL sensitivity to number phrase composition was observed. These results challenge purely syntactic hypotheses of the LATL and instead show that this region can be semantically modulated by the conceptual type of the composing element.

## Conflict of Interest Statement

The authors declare that the research was conducted in the absence of any commercial or financial relationships that could be construed as a potential conflict of interest.
